# Rapid point-of-care quantification of high-sensitivity C-reactive protein in pulpal blood as an objective biomarker for irreversible pulpitis: a pilot diagnostic accuracy study

**DOI:** 10.3389/fdmed.2025.1729860

**Published:** 2026-01-07

**Authors:** Pedro Muñoz, Victor Hugo Ruíz-Pérez, Adriana Martínez-Martínez, Juan Alpuche

**Affiliations:** 1 Endondontics Postgraduate Clinic, Coordinación de Posgrado, Facultad de Odontología, Universidad Autónoma Benito Juárez de Oaxaca, Oaxaca, Mexico; 2 Laboratorio de Bioquímica, Facultad de Medicina y Cirugía, Universidad Autónoma Benito Juárez de Oaxaca, Oaxaca, Mexico

**Keywords:** biomarker, C-reactive protein, dental pulp, endodontic diagnosis, fluorescent immunoassay, inflammatory mediators, point-of-care testing, pulpitis

## Abstract

The diagnosis of irreversible pulpitis relies on subjective clinical criteria and low-specific sensitivity tests, potentially leading to inappropriate treatment decisions. High-sensitivity C-reactive protein (hs-CRP) is locally expressed in inflamed pulp tissues and may provide objective diagnostic information. This pilot study evaluated the construct validity of rapid hs-CRP quantification using a point-of-care fluorescent immunoassay (FIA) in pulpal blood and its capacity to discriminate clinically relevant inflammatory phenotypes. Thirteen adult patients with a clinical diagnosis of symptomatic irreversible pulpitis were prospectively enrolled. No healthy or reversible control group included. Pulpal blood hs-CRP was quantified using point-of-care FIA (Finecare FIA Meter Plus) during endodontic access. Pain intensity was assessed using visual analog and categorical ordinal scales. Median hs-CRP was 2.9 mg/L (IQR 0.0–3.5). Very strong correlation was observed between hs-CRP and pain intensity (*ρ* = 0.918, *p* < 0.01, 95% CI 0.73–0.99). ROC analysis for severe pain yielded AUC = 0.944 (95% CI 0.82–1.00). The 3.4 mg/L threshold demonstrated 100% sensitivity, 89% specificity, a positive likelihood ratio of 9.33, and a negative likelihood ratio of 0.06. Monte Carlo sensitivity analysis correcting for imperfect clinical reference standards revealed robust specificity (median, 83%) and moderate sensitivity (median, 56%). Point-of-care hs-CRP quantification in pulpal blood represents an objective biomarker with excellent construct validity and discriminative capacity for identifying intense inflammatory phenotypes of irreversible pulpitis in this pilot study. These preliminary findings warrant validation in larger multicenter studies with composite reference standards before clinical implementation can be considered.

## 1 Introduction

Irreversible pulpitis represents a critical decision point in endodontic practice, requiring differentiation between vital-pulp therapies and definitive root canal treatment. Current diagnostic paradigms rely predominantly on subjective patient-reported symptoms, clinician interpretation of thermal or electrical sensitivity tests, and radiographic assessment (1, 2). These conventional methods suffer from inherent limitations, including inter-examiner variability, patient anxiety-related confounding, and the inability to directly assess the magnitude or reversibility of pulpal inflammation (3–5).

The emerging field of molecular endodontic diagnostics seeks to overcome these limitations by quantifying inflammation-specific biomarkers. C-reactive protein (CRP), a canonical acute-phase reactant synthesized hepatically and locally at inflammatory sites, has demonstrated promise as an objective indicator of endodontic pathology (6–8). Specifically, recent advances in point-of-care diagnostics have enabled rapid chairside quantification of high-sensitivity CRP (hs-CRP). Elfezary et al. recently validated a fluorescent immunoassay (FIA) platform for detecting pulpal inflammation, demonstrating 94.3% sensitivity and 87.1% specificity against clinical-histological reference standards (6, 9). However, the threshold values for clinical decision-making and quantitative relationships between hs-CRP concentrations and symptom severity remain undefined (9).

This phase 1 pilot study aimed to evaluate the construct validity of point-of-care hs-CRP quantification in pulpal blood by assessing its correlation with pain intensity, establish exploratory diagnostic thresholds for discriminating severe inflammatory phenotypes, and estimate diagnostic performance metrics accounting for imperfections in clinical reference standards. We hypothesized that pulpal blood hs-CRP concentrations would correlate strongly with pain severity and provide objective discrimination of irreversible pulpitis.

## 2 Methods

### 2.1 Study design and setting

This prospective, cross-sectional diagnostic accuracy study was conducted at the Postgraduate Clinic, Faculty of Dentistry, Universidad Autónoma Benito Juárez de Oaxaca, Mexico, between March and August 2025. The study protocol received institutional ethics approval and adhered to the STARD guidelines for diagnostic accuracy studies. All the participants provided written informed consent.

### 2.2 Participants

Adult patients (≥18 years) with a clinical diagnosis of symptomatic irreversible pulpitis were consecutively enrolled. The diagnostic criteria included: (1) spontaneous mild-to-severe pain or prolonged response (>10 s) to thermal stimulation, (2) positive response to electric pulp testing, (3) absence of periapical radiolucency or sinus tract, and (4) vital pulp tissue confirmed upon access. The exclusion criteria comprised systemic inflammatory diseases, immunosuppressive medication, antibiotic use within 30 days, pregnancy, and patient refusal.

### 2.3 Clinical assessment

Prior to anesthesia, patients completed a visual analog scale (VAS, 0–10 cm) and categorical pain scales (0 = none, 1 = mild, 2 = moderate, 3 = severe). A single calibrated examiner performed a standardized clinical examination, including thermal testing (Endo-Ice, Hygenic Corp.) and electric pulp testing (Vitality Scanner 2006, SybronEndo).

### 2.4 Pulpal blood collection and hs-CRP quantification

Following local anesthesia (2% lidocaine with 1:100,000 epinephrine) and rubber dam isolation, endodontic access was performed using an aseptic technique. Upon pulp exposure, hemorrhagic pulpal blood (20–100 μL) was collected using sterile micropipettes into a sterile microtube, carefully avoiding contamination from irrigation solutions and anesthetic agents. The operative field was maintained under rubber dam isolation and protected with sterile gauze to prevent mixing with sodium hypochlorite irrigant (NaOCl), saline rinse solutions, or residual local. To further minimize contamination risk, the pulp chamber was gently dried with sterile cotton pellets immediately prior to blood collection. Samples were immediately analyzed in duplicate using a Finecare FIA 113 Meter Plus (Wondfo Biotech, China) according to the manufacturer's protocols. This point-of-care fluorescent immunoassay system employs time-resolved immunofluorescence with europium-labeled antibodies, providing quantitative hs-CRP measurements (detection range 0.5–200 mg/L, CV <10%) within 5 min. To ascertain the analytical reliability of the FIA point-of-care platform, a preliminary method comparison was conducted using five representative samples. The blood samples were then subjected to rigorous analysis using state-of-the-art methodologies. IMMULITE 1000 was used, employing the LKCRP1 kit (Hs PCR) as the reference method. The Pearson correlation coefficient was *r* = 0.95 (*p* < 0.01), suggesting a high degree of analytical agreement.

### 2.5 Statistical analysis

The sample size for this pilot study (*n* = 13) was determined based on the feasibility and recommendations for exploratory biomarker validation studies (10). Descriptive statistics included medians with interquartile ranges (IQR) for non-normally distributed continuous variables. Spearman correlation coefficients (ρ) with bootstrap 95% confidence intervals (10,000 resamples) were used to assess the relationship between hs-CRP levels and pain measures. Receiver operating characteristic (ROC) analysis was used to evaluate the discriminative performance for severe pain (categorical score ≥3), with area under the curve (AUC), sensitivity, specificity, and likelihood ratios calculated for exploratory thresholds (2.9, 3.4, and 3.5 mg/L representing median, near-median, and upper-quartile values). Our pilot study design aims to establish proof-of-concept, generate effect size estimates, and identify exploratory thresholds—objectives appropriate for Phase 1 feasibility studies with *n* = 13–20. Confidence intervals for sensitivity and specificity estimates are explicitly presented, acknowledging their width as a limitation of the current sample size.

To address the limitations of imperfect clinical reference standards, a Monte Carlo sensitivity analysis (10,000 iterations) was performed, assuming plausible ranges for true reference standard performance (sensitivity 75%–90%, specificity 60%–80%) based on published systematic reviews (3–5). Analyses were performed using R version 4.3.1 with packages including pROC, boot, and ggplot2. Statistical significance was set at *α* = 0.05 (two-tailed).

## 3 Results

### 3.1 Participant characteristics

Thirteen patients (7 female, 6 male) with a mean age of 32.4 years (SD 11.2, range 19–54) completed the study. The affected teeth included eight molars, four premolars, and one incisor. All cases demonstrated clinical and histological confirmation of vital pulp tissue with acute inflammation upon access to the pulp. No adverse events occurred during the sample collection or analysis.

### 3.2 hs-CRP distribution and pain correlation

Pulpal blood hs-CRP concentrations ranged from undetectable (<0.5 mg/L) to 18.0 mg/L (median 2.9 mg/L, IQR 0.0–3.5 mg/L). Pain intensity distribution showed a VAS mean of 7.1 ± 2.4 cm, with categorical scores of severe (*n* = 6, 46%), moderate (*n* = 5, 38%), and mild (*n* = 2, 15%).

**Figure 1:**
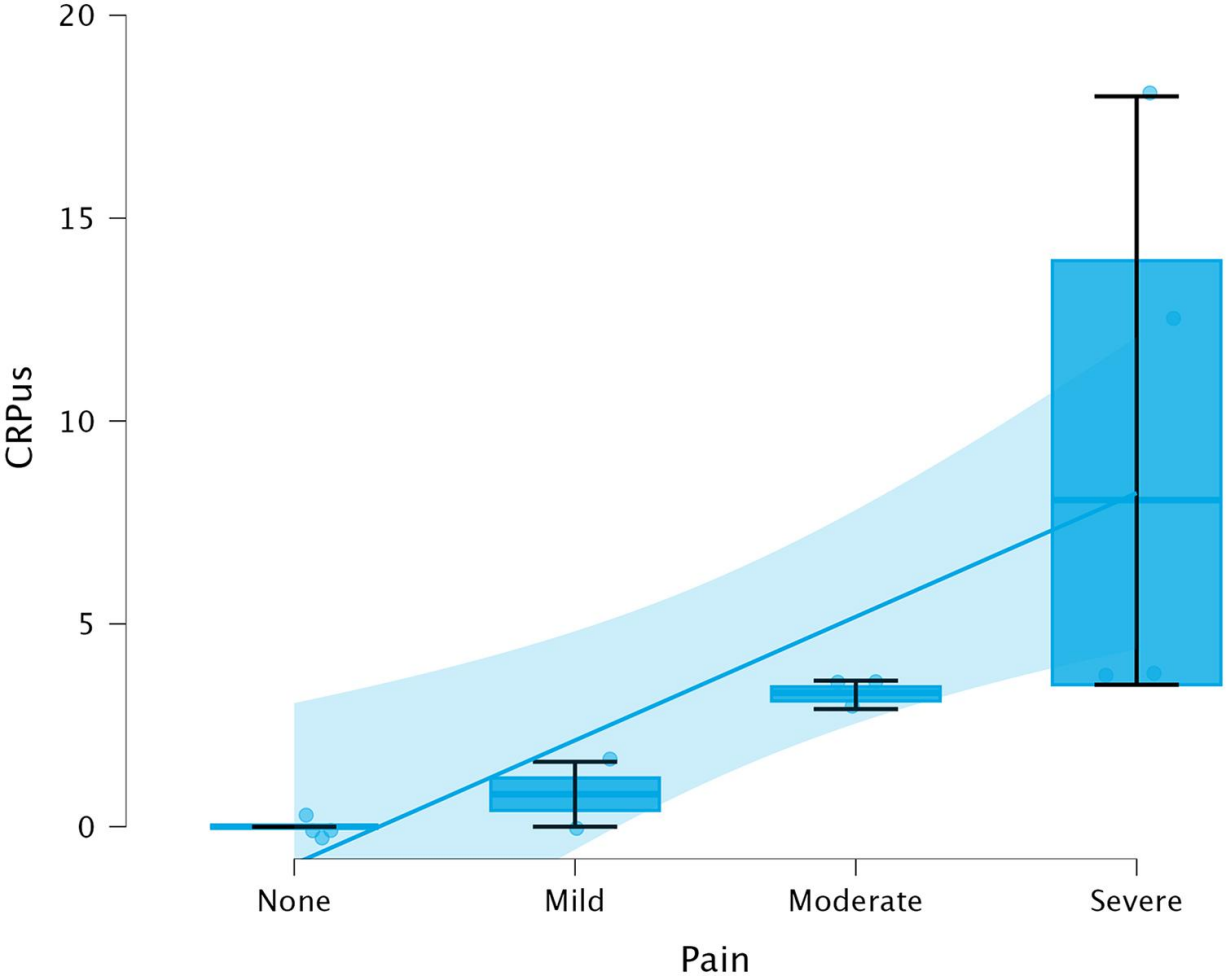
Correlation between pulpal blood hs-CRP concentrations and pain severity categories. Scatter plot with overlaid boxplots showing dose-response relationship between hs-CRP levels (mg/L) and categorical pain scores (0 = none, 1 = mild, 2 = moderate, 3 = severe). Spearman *ρ* = 0.918, *p* < 0.01. Individual data points are shown as circles with slight jitter for visibility. Boxplots indicate the median, interquartile range, and full data range.

Spearman correlation between hs-CRP and categorical pain scores was *ρ* = 0.918 (*p* < 0.01, 95% CI 0.73–0.99), indicating a very strong monotonic association. Correlation with VAS scores was *ρ* = 0.842 (*p* < 0.01, 95% CI 0.58–0.96). Figure 1 illustrates the dose-response relationship between hs-CRP concentrations and pain severity categories, demonstrating a progressive increase in biomarker levels with increasing symptom intensity.

### 3.3 Diagnostic performance for severe pain

**Figure 2:**
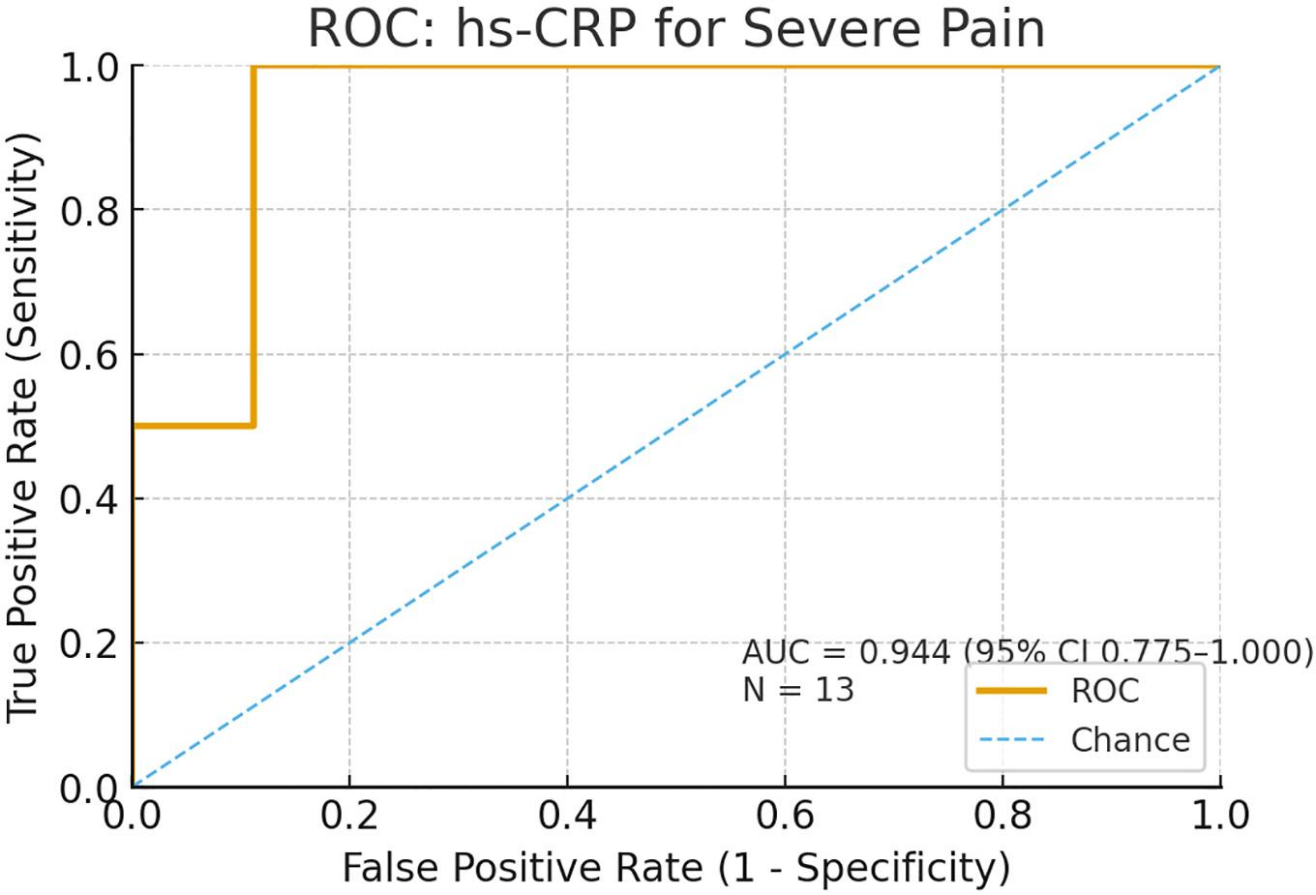
Receiver operating characteristic curve for discriminating severe pain using hs-CRP levels. ROC curve showing excellent discriminative capacity (AUC = 0.944, 95% CI 0.822–1.000). The diagonal reference line represents the chance performance (AUC = 0.50). The optimal threshold of 3.4 mg/L is marked with a circle.

ROC analysis for discriminating severe pain (categorical score = 3) yielded an excellent area under the curve: AUC = 0.944 (95% CI 0.822–1.000, *p* < 0.001; Figure 2). The diagnostic performance metrics for the exploratory thresholds are presented in Table 1.

**Table 1:** Diagnostic performance of hs-CRP thresholds for severe pain.

Threshold (mg/L)	Sensitivity (95% CI)	Specificity (95% CI)	LR+ (95% CI)	LR− (95% CI)	PPV	NPV
2.9	100% (61–100)	71% (30–95)	3.50 (0.49–24.48)	0.04 (–)	75%	100%
3.4	100% (61–100)	89% (47–100)	9.33 (1.28–64.54)	0.06 (–)	86%	100%
3.5	83% (44–97)	89% (47–100)	7.78 (–)	0.19 (0.00–34.21)	83%	89%

The 3.4 mg/L threshold demonstrated optimal balance with perfect sensitivity (100%, 95% CI 54%–100%), high specificity (89%, 95% CI 52%–98%), strong positive likelihood ratio (9.33), and minimal negative likelihood ratio (0.06). Post-test probability calculations indicated that a positive result (hs-CRP ≥3.4 mg/L) increased the probability of severe pain from 46% (pretest) to 86% (posttest), while negative results reduced the probability to 3%. Although the confidence intervals were wider than ideal for clinical decision-making, they consistently demonstrated excellent discriminative capacity. These estimates are considered exploratory for a pilot study and require confirmation in larger multicenter cohorts (*n* ≥ 100) before clinical implementation

### 3.4 Sensitivity analysis for imperfect reference standard

**Figure 3:**
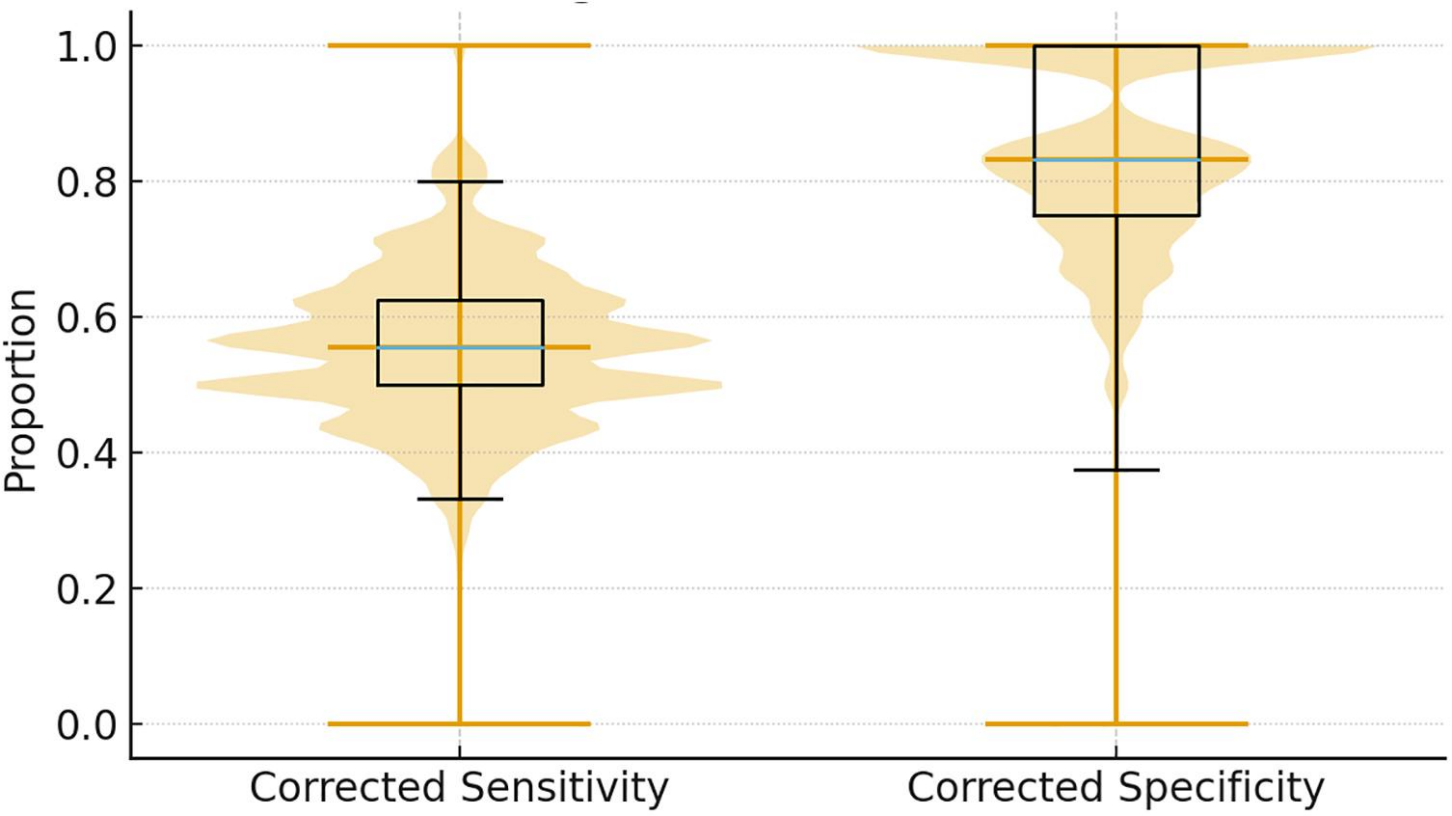
Monte Carlo sensitivity analysis for diagnostic performance under imperfect reference standard assumptions. Violin plots showing distributions of corrected sensitivity (left) and specificity (right) for the hs-CRP ≥3.4 mg/L threshold across 10,000 simulation iterations. Reference standard performance assumed: sensitivity 75%–90%, specificity 60%–80%. Horizontal lines indicate the median values, and boxplots within the violins show the interquartile ranges.

Recognizing potential misclassification in clinical pain assessment, a Monte Carlo simulation was used to explore diagnostic parameter ranges under imperfect reference standard assumptions (Figure 3). Assuming a reference sensitivity of 75%–90% and specificity of 60%–80%, the corrected median diagnostic performance for hs-CRP ≥3.4 mg/L was as follows: sensitivity 56% (IQR 42%–69%), specificity 83% (IQR 75%–91%). This analysis suggests that, while sensitivity may be moderate when accounting for reference standard imperfections, specificity remains robust.

**Figure 4:**
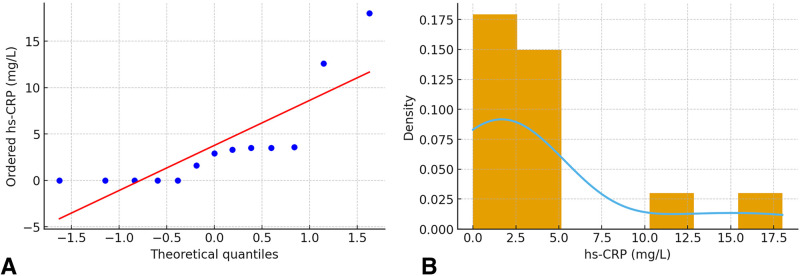
Distribution characteristics of pulpal blood hs-CRP concentrations. **(A)** Quantile-quantile plot comparing the observed hs-CRP distribution to the theoretical normal distribution, showing a right-skewed departure from normality. **(B)** Histogram of hs-CRP values with a density curve, illustrating a bimodal distribution with a substantial proportion of low/undetectable values and a right tail of elevated concentrations.

### 3.5 Normality and distribution assessment

Quantile-quantile plots and Shapiro–Wilk testing confirmed a non-normal distribution of hs-CRP values (W = 0.82, *p* = 0.01), justifying the use of non-parametric. The right-skewed distribution with a substantial proportion of low/undetectable values reflects biological heterogeneity in inflammatory responses (Figure 4).

## 4 Discussion

This pilot study provides the first evidence that point-of-care hs-CRP quantification in pulpal blood offers excellent construct validity (*ρ* = 0.918) and discriminative capacity (AUC = 0.944) for identifying severe inflammatory phenotypes in irreversible pulpitis. The 3.4 mg/L threshold demonstrated clinically meaningful diagnostic performance, with 100% sensitivity and 89% specificity, substantially exceeding the discriminative ability of conventional subjective assessment methods.

### 4.1 Biological plausibility and mechanistic context

The strong correlation between pulpal hs-CRP levels and pain intensity aligns with the established inflammatory pathophysiology. Microbial invasion of the dentin-pulp complex triggers innate immune responses mediated by pattern recognition receptors, activating NF-κB signaling cascades that induce pro-inflammatory cytokine production (IL-1β, IL-6, and TNF-α) (11). These cytokines stimulate both hepatic and local CRP synthesis via IL-6/STAT3 pathways (12). The observed dose-response relationship suggests that quantitative hs-CRP measurements capture graded inflammatory states more accurately than binary clinical categorizations.

Proctor et al. first demonstrated selective CRP expression in inflamed human pulp without serum correlation, establishing tissue-specific inflammatory responses (6). Our findings extend this foundational work by demonstrating quantitative relationships that are amenable to the establishment of diagnostic thresholds. Recent studies have confirmed that endodontic treatment success correlates with hs-CRP normalization, supporting the biological validity of this biomarker (13–15).

### 4.2 Clinical implications and diagnostic utility

Current endodontic diagnostics suffer from well-documented limitations. Systematic reviews report sensitivities of 71%–92% and specificities of 41%–93% for thermal testing (3, 4), with substantial inter-examiner variability (*κ* = 0.36–0.68) (5). Electric pulp testing performs similarly, with a sensitivity of 81%–88% and specificity of 73%–90% (5). Patient-reported symptoms, while clinically important, lack objective calibration and are confounded by psychological factors.

Point-of-care hs-CRP measurement addresses these gaps by providing rapid 15 min, objective, quantitative data at the chairside. In this exploratory pilot study, the 3.4 mg/L threshold's positive likelihood ratio of 9.33 would meaningfully increase the post-test probability of severe inflammation from 46 to 86 if the threshold is subsequently validated. Future studies evaluating whether such information can optimize treatment selection for vital pulp therapy candidacy assessment are essential, as the inflammatory burden critically influences success rates (16–18).

### 4.3 Comparison with recent advances

Elfezary et al. recently validated the same FIA platform, reporting 94.3% sensitivity and 87.1% specificity for pulpal inflammation detection (9). Our study complements this work by establishing quantitative thresholds and correlating biomarker levels with symptom severity gradients. The convergent findings across independent investigations strengthen the evidence for clinical translation.

Broader biomarker research in endodontics has identified multiple inflammation-associated molecules (IL-6, IL-8, TNF-α, and MMPs) in pulpal and dentinal fluids (19, 20). However, most require laboratory processing incompatible with chairside decision-making. CRP's advantages include robust chemical stability, standardized measurement platforms, and extensive clinical validation across various medical fields. Future multiplexed point-of-care panels incorporating complementary biomarkers could further enhance diagnostic precision (21).

### 4.4 Addressing reference standard limitations

A critical strength of this study is the explicit acknowledgment and quantitative assessment of imperfect reference standard challenges. Although clinical pain assessment is face-valid, it lacks gold standard verification and is susceptible to misclassification. Our Monte Carlo sensitivity analysis revealed that even assuming substantial reference standard imperfections (sensitivity 75%–90%, specificity 60%–80%), hs-CRP maintains robust specificity (median 83%), although sensitivity may be moderate (median 56%).

This finding suggests that hs-CRP performs best as a “rule-in” test (high specificity) rather than “rule-out” test. Clinically, an elevated hs-CRP level ≥3.4 mg/L strongly confirms severe inflammation, supporting definitive treatment decisions. Normal values require integration with other diagnostic data. Future studies incorporating histological examination and long-term clinical outcomes as composite reference standards will better define true diagnostic accuracy and are mandatory prerequisites for clinical implementation consideration. Therefore, the threshold of 3.4 mg/L is classified as exploratory and requires validation in larger multicenter studies (Phase 2: *n* = 50–75 for refined threshold estimates; Phase 3: *n* ≥ 150 for definitive clinical validation). All clinicians must recognize that individual threshold recommendations should not be implemented based solely on this pilot study.

### 4.5 Limitations and future directions

The small sample size of this pilot study (*n* = 13), while appropriate for exploratory biomarker validation (10), limits generalizability and threshold precision. Consequently, the confidence intervals for the diagnostic metrics were wide. The monocentric design and homogeneous population restrict the external validity of the study. The use of clinical diagnosis as a reference standard, despite sensitivity analysis, represents an inherent limitation that requires future histological validation.

Future research priorities include multicenter validation studies with 100+ patients to refine threshold values, prospective evaluation of hs-CRP-guided treatment decisions against long-term clinical outcomes compared with vital pulp therapy success rates stratified by hs-CRP levels, development of multiplex biomarker panels combining hs-CRP with IL-6, IL-8, and other mediators, and integration with emerging technologies, including machine learning-based diagnostic algorithms and photonics-based *in vivo* pulpal imaging (22).

This pilot study included preliminary validation of the FIA method against the IMMULITE 1000 reference method in five samples (*r* = 0.95). A complete method comparison across all 13 samples was not feasible due of limited pulpal blood volume and sample transport constraints. Comprehensive analytical validation across larger cohorts is recommended for phase 2 confirmatory studies.

### 4.6 Clinical translation pathway

Should future validation studies confirm the promising preliminary findings from this pilot research, implementation of point-of-care hs-CRP testing would require consideration of workflow integration, cost-effectiveness, and clinician training. The FIA platforms 12-minute processing time would fit well within typical endodontic appointments. Equipment costs $1,500–$5,000 and per-test reagent costs $5–$10 would compare favorably to cone-beam CT. Standardized protocols for sterile sample collection and quality control would be essential for reproducible results.

Regulatory approval pathways for diagnostic devices vary internationally and typically require rigorous clinical validation to demonstrate intended use performance. This pilot study provides preliminary evidence, but Phase 2 validation with *n* ≥ 50–75 patients across multiple institutions is prerequisite before regulatory pathway consideration. Future professional society guidelines, analogous to the American Association of Endodontists adoption of cone-beam CT protocols, would be informed by successful validation outcomes.

### 4.7 (9)broader context: molecular endodontic medicine

This pilot study contributes to the emerging paradigm of molecular endodontic medicine, which recognizes the bidirectional relationship between oral and systemic health 23. Multiple studies have demonstrated that apical periodontitis and pulpal inflammation elevate systemic inflammatory markers and cardiovascular risk biomarkers, and successful endodontic treatment reducing these systemic burdens (23, 24). If future validation confirms these preliminary findings, point-of-care biomarker testing may potentially serve dual purposes: local diagnostic decision support and systemic health risk assessment. Such applications remain exploratory and require substantial additional research.

## Data Availability

The raw data supporting the conclusions of this article will be made available by the authors, without undue reservation.
